# Early active normothermia versus no temperature control in a porcine model of extracorporeal cardiopulmonary resuscitation

**DOI:** 10.1186/s40635-026-00958-6

**Published:** 2026-08-05

**Authors:** Quentin de Roux, Rébecca Goutchtat, Fanny Lidouren, Ali Jendoubi, Naoto Watanabe, Nadir Mouri, Luiza Bouchet, Matthias Kohlhauer, Bijan Ghaleh, Alice Hutin, Stéphane Germain, Nicolas Brechot, Nicolas Mongardon, Renaud Tissier

**Affiliations:** 1 Ecole nationale vétérinaire d’Alfort, IMRB, AfterROSC Network, Maisons- Alfort, F-94700 France; 2 Service d’anesthésie-réanimation et médecine péri-opératoire, Hôpitaux Universitaires Henri Mondor, Assistance Publique-Hôpitaux de Paris (AP-HP), Créteil, F-94010 France; 3 GRCT OPTIMA, Université Paris Est Créteil, Hôpitaux Universitaires Henri Mondor, Créteil, F- 94010 France; 4 Univ Paris Est Créteil, INSERM, IMRB, Créteil, F-94010 France; 5 Département de biochimie-pharmacologie-biologie moléculaire-génétique médicale, Hôpitaux Universitaires Henri Mondor, Assistance Publique-Hôpitaux de Paris (AP-HP), Créteil, F-94700 France; 6 SAMU de Paris, Hôpital Necker, Assistance publique-Hôpitaux de Paris (AP-HP), Paris, F- 75015 France; 7 Médecine intensive-Réanimation, Hôpital Européen Georges Pompidou, Assistance Publique-Hôpitaux de Paris (AP-HP), Paris, F-75015 France

**Keywords:** Cardiac arrest, Resuscitation, Extracorporeal cardiopulmonary resuscitation, Shock, Targeted temperature management

## Abstract

**Aim:**

Early downward temperature drift is a common event of pre-hospital extracorporeal cardiopulmonary resuscitation (e-CPR), since prehospital extracorporeal membrane oxygenation (VA-ECMO) circuits often lack heat-exchangers. However, whether this core body temperature evolution could be detrimental or beneficial compared to strict normothermia in this context remains unclear. Accordingly, in a swine model of refractory cardiac arrest, we compared e-CPR with heat-exchanger-assisted active maintenance of normothermia versus e-CPR with no temperature regulating device.

**Methods and results:**

Female pigs underwent ventricular fibrillation with 15 min of no-flow and subsequent e-CPR using VA-ECMO. Animals were randomized (*n* = 6 per group) to receive either active normothermia (with heat-exchanger connected to the VA-ECMO circuit) or no temperature control (without heat-exchanger). Fluids and vasopressor requirements, hemodynamics, ECMO parameters, blood gases, and ultra early impact on target organs biomarkers were monitored for 120 min after return of spontaneous beating (ROSB). Core temperature was significantly lower in the group without heat-exchanger use (36.1 ± 0.3 vs. 37.5 ± 0.3 °C, *p* = 0.0002). However, fluid requirements, vasopressor doses, heart rate, arterial pressure, intracranial pressure and ECMO flow were similar between groups. Blood gases and biomarkers (including troponin I, creatinine, ALAT and Protein S100β) showed no relevant differences.

**Conclusions:**

Withholding active temperature regulation during early VA-ECMO after refractory cardiac arrest resulted in lower core temperatures but did not significantly affect macrohemodynamics, ECMO flow, fluids/vasopressor needs, or ultra early impact on target organs biomarkers. The short use of simplified ECMO circuits without active temperature management does not appear to result in severe hypothermia or major early hemodynamic instability over a clinically realistic transport like timeframe.

## Introduction

In cases of refractory cardiac arrest, extracorporeal cardiopulmonary resuscitation (e-CPR) can be considered as a rescue therapy when conventional CPR fails [1, 2]. It has been associated with survival rates of approximately 25% with good neurological outcomes [3–5] but e-CPR remains associated with severe shock and post-resuscitation multi-organ failure [6]. Accordingly, several strategies, such as targeted temperature management, have been proposed to improve post-cardiac arrest outcomes. Indeed, core body temperature reduction may offer neuroprotection by limiting ischemia-reperfusion injury [7, 8]. However, after conventional CPR, moderate spontaneous hypothermia failed to provide significant benefits compared with strict normothermia [9].

In routine practice, most prehospital e-CPR patients present mild downward temperature drift because veno-arterial extracorporeal membrane oxygenation (VA-ECMO) devices lack heat-exchangers due to space and logistic constraints. For instance, patients were shown to arrive in the intensive care unit at 33–34° [10, 11], with subsequent rewarming or not, depending on institutional protocols.

Therefore, if the active control of temperature is usually considered as essential [12], early downward temperature drift benefits or risk during e-CPR remains questionable. To address this issue, we aimed at evaluating whether actively maintaining normothermia with a heat-exchanger during the early phase of e-CPR as compared with absence of heat-exchanger on VA-ECMO circuit, change hemodynamic parameters in a swine model of refractory cardiac arrest.

## Materials and methods

All experiments were conducted in accordance with French regulations governing animal research. The experimental protocol was approved by the institutional animal care and use committee (APAFIS #38967–2022102416497377 v6).

### Animal preparation

After their arrival, female domestic pigs (Lebeau, Gambais, France) benefited from a 10-day acclimatization period in collective housing that met all guidelines for animal research (floor space, temperature, enrichment, and free access to water and food).

As previously described [13–16], swine (35–37 kg) were premedicated using a mixture of zolazepam/tiletamine (10 mg/kg i.m.) and methadone chlorhydrate (0.75 mg/kg i.m.). After anesthesia with propofol (2.5 mg/kg i.v), tracheal intubation was performed and mechanical ventilation started with inspired fraction of oxygen set at 30%, tidal volume at 8 ml/kg and respiratory rate adjusted to target end-tidal capnia between 35 and 45 mmHg. Anesthesia was maintained with sevoflurane (Baxter^®^, Illinois, USA) with 1–2% expiration target. No neuromuscular blocking agent was used. Several catheters were inserted using ultrasound: one into the femoral artery (7-French catheter), one into the femoral vein (9-French catheter) and one into the right external jugular vein (7-French triple‐lumen catheter, Arrow International, Reading, PA, USA) for intravenous treatment administration. Arterial pressure was monitored with hydraulic tube placed in aortic arch thanks to catheter inserted in femoral artery. After left cervical incision, a 3 mm diameter flow probe was placed around the carotid artery to continuously measure carotid blood flow (PS–Series Probes, Transonic^®^, NY, USA). An intracranial pressure catheter was positioned to continuously monitor intracranial pressure (Millar^®^, SPR-524, Houston, TX, USA) and a bladder catheter was surgically inserted. All parameters were continuously recorded throughout the experimental protocol (HEM version 4.4, Notocord, Croissy-sur-Seine, France). After instrumentation, infusion of lactated Ringer’s solution (Ringer Lactate, CEVA, France) 15 mL/kg i.v. and unfractionated heparin bolus (100 IU/kg i.v.) were administered to achieve correct anticoagulation before ECMO implantation. Bladder was emptied before cardiac arrest, and vesical temperature was continuously measured.

### Experimental protocol of cardiac arrest and cardiopulmonary resuscitation

**Fig. 1:**
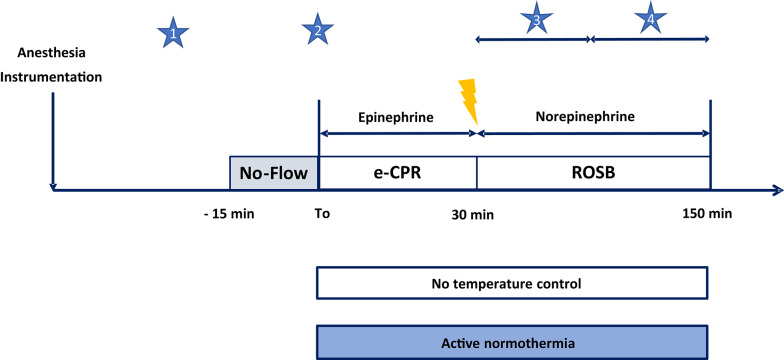
Experimental protocol Points 1 and 2 correspond to boluses of Ringer’s lactate (15 ml/kg, i.v.), and points 3 and 4 indicate the maximum amount of fluids allowed per hour (30 ml/kg/h, i.v.). The lightning symbol represents the external electrical shock and the return to sinus rhythm. Animals were euthanized for 150 min. n = 6 per group. Data are presented as mean ± SEM for continuous variables. * p < 0.05 for the group x time interaction effect. e-CPR: extracorporeal cardiopulmonary resuscitation; ROSB: return of spontaneous beating

The experimental protocol is summarized in Fig. 1. A stabilization period of 30 min was considered between the initial phase of instrumentation and the induction of cardiac arrest. Sevoflurane inhalation was stopped, and cardiac arrest was induced by an alternating current of 10 V with a pacemaker catheter into the right ventricle, using catheter inserted into the femoral vein. Mechanical ventilation stopped after ventricular fibrillation onset. During the 15-min no-flow period, venous (21 Fr, 55 cm) and arterial (15 Fr, 23 cm) cannulas (BioMedicus, Medtronic, Inc., USA) were inserted into the femoral vessels and connected to a shortened (330 mL) Medos VA-ECMO circuit (Medizintechnik, Stolberg, Germany).

e-CPR was initiated using femoro-femoral VA-ECMO and maintained for 30 min (low-flow phase). ECMO blood flow and gas blender flow were both set at 40 mL/kg/min. This flow rate was selected as the highest possible flow rate for animals of this size. FiO₂ and FmO₂ were kept identical and adjusted to maintain arterial PaO₂ between 80 and 100 mmHg. Immediately after ECMO initiation, animals received an additional infusion of lactated Ringer’s solution (15 mL/kg i.v.). Epinephrine infusion was titrated to maintain a mean arterial pressure (MAP) of 65–70 mmHg for 30 min (with a maximum dose of 5.0 µg/kg/min).

After 30 min of e-CPR, electric shocks (150 J) were delivered to achieve return of spontaneous beating (ROSB) and epinephrine infusion was then stopped. ROSB was defined as return of spontaneous beating with cardiac assistance. Norepinephrine was added to achieve the MAP target, with a maximum dose of 5.0 µg/kg/min. At the same time, mechanical ventilation was restarted with lung protective settings (tidal volume 4 mL/kg, respiratory rate 20 cycles per minute, positive end-expiratory pressure 5 cm H_2_O). In case of concomitant arterial hypotension (MAP < 65 mmHg) and ECMO flow drop (< 40 ml/kg/min despite the increase in the number of rotations per minute) and with maximal fluid and norepinephrine administration, no additional treatment was attempted. Conversely, if the hemodynamic status was favorable with MAP spontaneously > 80 mmHg (i.e. without catecholamine), a stepwise reduction of ECMO flow by 10 ml/kg/min was attempted every 15 min. Lactated Ringer’s solution was administered at a maximum amount of 30 ml/kg/h to maintain ECMO flow target. At the end of the protocol, swine were euthanized with pentobarbital (60 mg/kg i.v.).

### Experimental groups

On the morning of the experiment, a team member (not directly involved in the project) blindly and randomly assigned animals to groups with or without a heat exchanger. Due to the experimental design, blinding was not possible during the experiment. However, data collection, biological analyses and statistical analyses were conducted blindly to group allocation.

In active normothermia group (with heat-exchanger), the goal was to try to maintain a stable core body temperature of 38 ± 0.5 °C. A water-based heating system using a double-boiler setup was connected to the oxygenator membrane via dedicated tubing, allowing warm water to circulate at a target temperature of 38 °C. Water temperature was continuously monitored and adjusted throughout the procedure to maintain stable thermal conditions. In the no temperature control group (i.e. control group, without heat-exchanger), animals were not actively cooled and only experienced spontaneous temperature drift; no temperature target was set.

### Clinical and biological parameters

Multiple parameters were collected throughout the experiment. Hemodynamic variables included heart rate, arterial pressure, right atrial pressure, cardiac output measured by transthoracic echocardiography, and vasopressor requirements (epinephrine and norepinephrine). Carotid blood flow and intracranial pressure were continuously monitored. Fluid administration, VA-ECMO blood flow, pump speed (rotations per minute), and ventilatory parameters were also recorded.

Blood samples were regularly collected to assess pH, O_2_ and CO_2_ partial pressures as for arterial lactate, glucose and other biochemical parameters (POC Cobas b 123, Roche^®^, Switzerland). Renal function was assessed by measuring creatinine levels, and liver injury was evaluated through serum AST (Aspartate Aminotransferase), ALT (Alanine Aminotransferase). Myocardial and neurologic injury markers including serum levels of troponin I and Protein S100 (Protein S100β) were measured.

### Statistical analysis

Our primary evaluation criterion was the fluid volume required to achieve the target ECMO flow and blood pressure during e-CPR. Based on calibration results, fluid volume in an animal without rewarming was 19 ml/kg/h. Assuming a 30% increase in fluid volume in the active normothermia group (with standard deviation of 2.5 ml/kg/h, α = 0.05 and 1-β = 0.8), the required sample size was 6 included subjects per group (BiostatTGV). Statistical analyses were conducted using GraphPad Prism^®^ software (Boston, USA).

Normality assumptions were checked using the Shapiro-Wilk test. Data were presented as mean ± SEM in graphics or effect size with 95% confidence intervals. Continuous variables involving repeated measures over time for each animal were compared between groups using a 2-way ANOVA (or mixed-model if missing values), followed by Holm-Sidak post-hoc test for multiple comparison if needed. These variables were compared considering group, time and group x time effect. Significant group and group x time effect were reported in data analysis. In case of group x time significant interaction, a comparison at a different time point was made using a Fisher’s LSD test. Time effect was not reported. For each animal, measures were performed once at each different time point. For the variables that do not involve repeated measurements over time, such as total catecholamines requirement, comparisons were made using the non-parametric Mann-Whitney test. Statistical significance was defined as *p* ≤ 0.05.

## Results

Twelve animals were included and equally allocated into two groups of six. One animal did not complete the full protocol and died prematurely at M120 in the active normothermia group but was included in the final analysis. Animals’ weights were similar in both groups (35.5 ± 1.7 kg in control group and 35.3 ± 1.9 kg in active normothermia group).

### Temperature

**Fig. 2:**
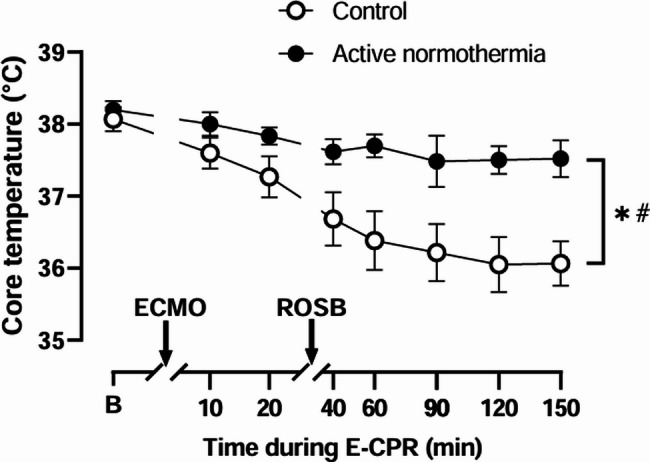
Core temperature. * p<0.05 for the group x time interaction effect and #, p<0.05 for the group effect with Mixed-effects analysis and Sidak post-hoc test. n = 6 per group. Continuous variables are expressed as mean ± SEM. e CPR: extracorporeal cardiopulmonary resuscitation; ROSB: return of spontaneous beating

All animals had an initial core temperature of 38.1 ± 0.1 °C. As shown in Fig. 2, core temperature decreased after cardiac arrest in both groups. In the active normothermia group, core temperature at the end of the follow-up was 37.5 ± 0.3 °C but remained within the target range. In the control group, core temperature was significantly lower at the end of the follow-up (36.1 ± 0.3 °C; with mean difference of −1.00 [−1.65; −0.22] °C *p* = 0.0002).

### Fluids and vasopressors requirement

**Fig. 3:**
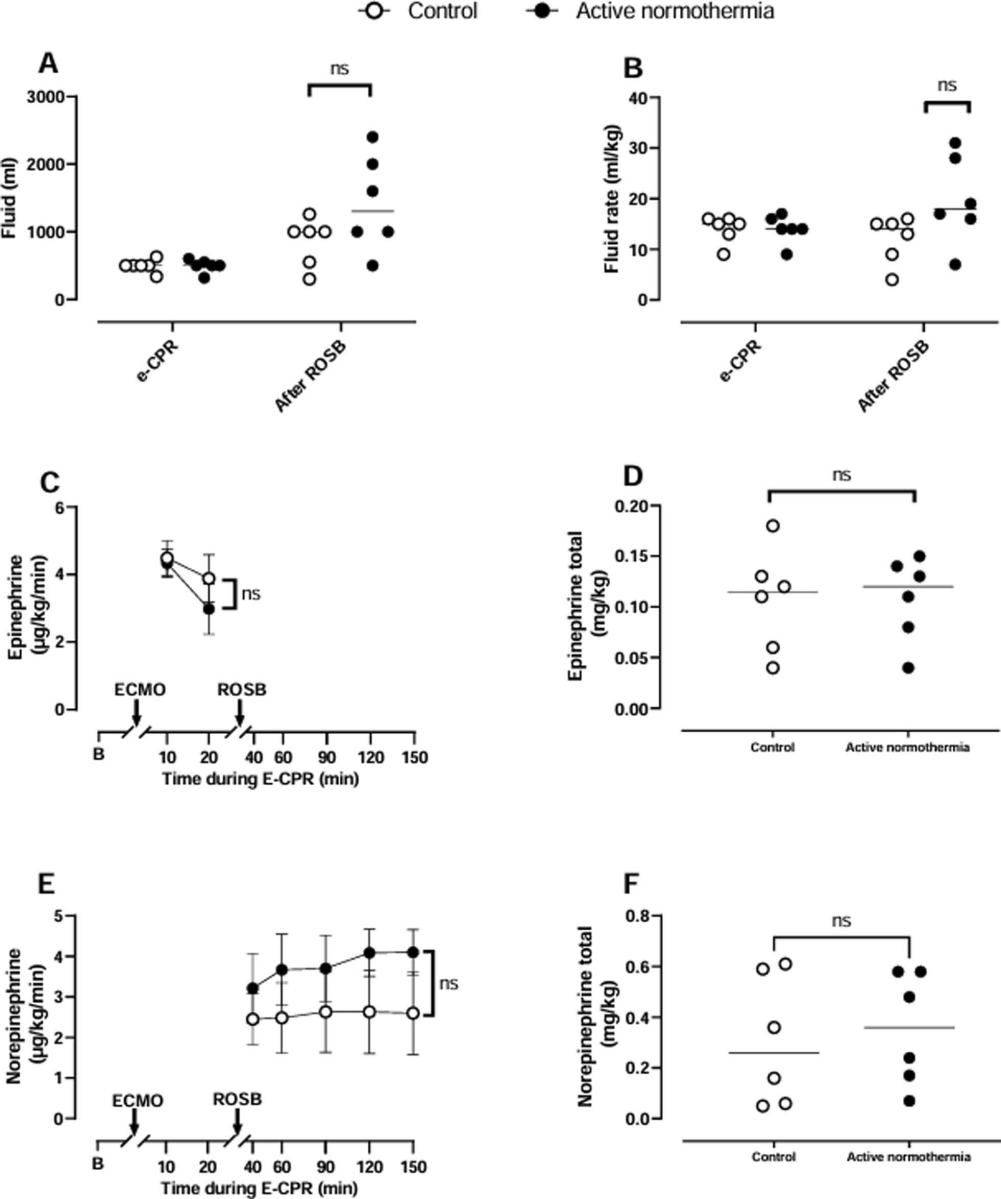
Vasopressor and fluid requirement (**A**: total fluid; **B**: fluid rate, **C**: norepinephrine, **D**: norepinephrine total, **E**: epinephrine, **F**: total epinephrine). n = 6 per group. Continuous variables are expressed as mean ± SEM. Each point indicates one animal for both the total catecholamine dose and the total amount of fluids administered. e CPR: extracorporeal cardiopulmonary resuscitation; ROSB: return of spontaneous beating

Fluids administered to achieve the targeted VA-ECMO flow did not differ significantly between groups throughout the protocol (16.22 ml/kg in the active normothermia group, versus 13.39 mL/kg in the control group, i.e. a difference between means of −2.83 [−6,46; 0.80] mL/kg *p* = 0.074). Norepinephrine requirements were not significantly different among groups after ROSB (Fig. 3).

### Hemodynamics and VA-ECMO parameters

**Fig. 4:**
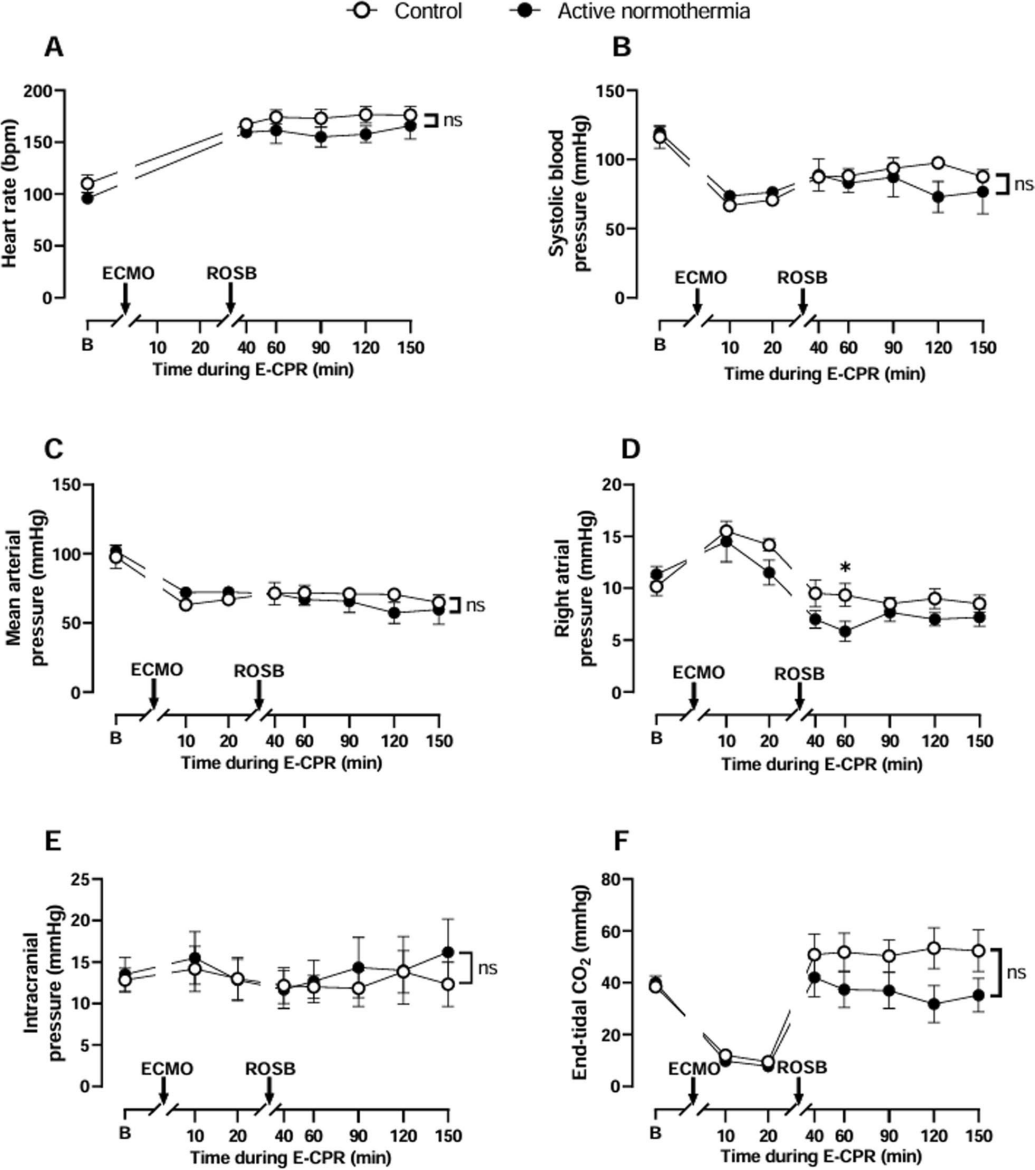
Hemodynamics and neurological parameters (**A**: heart rate, **B**: systolic arterial pressure, **C**: mean arterial pressure, **D**: right atrial pressure, **E**: intracranial pressure, **F**: end-tidal CO2). *, p<0.05 for the group x time interaction effect (p=0.04) with Mixed-effects analysis and Fisher’s LSD test. n = 6 per group. Continuous variables are expressed as mean ± SEM. e CPR: extracorporeal cardiopulmonary resuscitation; ROSB: return of spontaneous beating

As shown in Fig. 4, heart rate and arterial pressure were not different among groups at baseline and after cardiac arrest. During ventricular fibrillation, epinephrine required to maintain the MAP target was not significantly higher in the control group as compared to the other one. Right atrial pressure was higher at 60 min after cardiac arrest in the control group, as compared to other one (9.0 ± 1.0 vs. 6 ± 1.0 mmHg; *p* = 0.019 for the interaction time × group and *p* = 0.04 for 60 min, with a mean difference of 3.50 [0.18; 6.82] mmHg). Intra-cranial pressure was not different among groups.

**Fig. 5:**
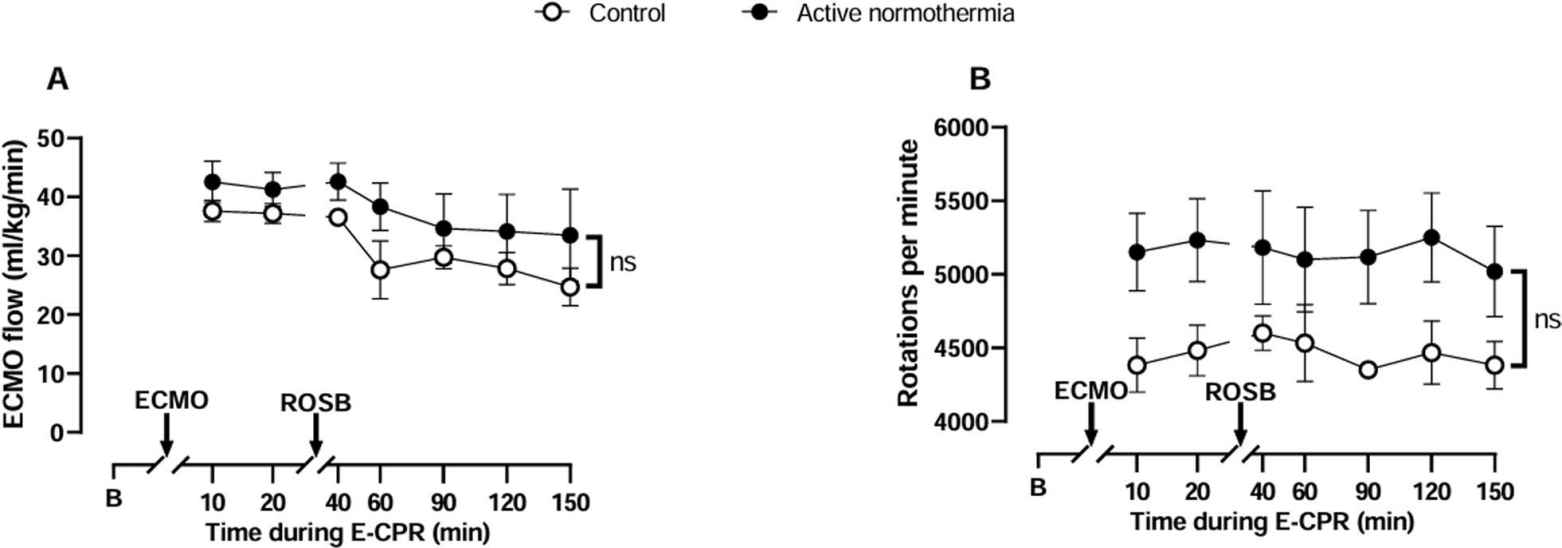
VA-ECMO parameters (**A**: ECMO flow; **B**: rotation per minute). n = 6 per group. Continuous variables are expressed as mean ± SEM. e CPR: extracorporeal cardiopulmonary resuscitation; ROSB: return of spontaneous beating

As illustrated in Fig. 5, VA-ECMO flow rates were not different between groups throughout the follow-up period. In the active normothermia group, maintaining the same flow required a non-significantly higher pump speed compared to the other group.

### Arterial blood gases and blood biochemistry

**Fig. 6:**
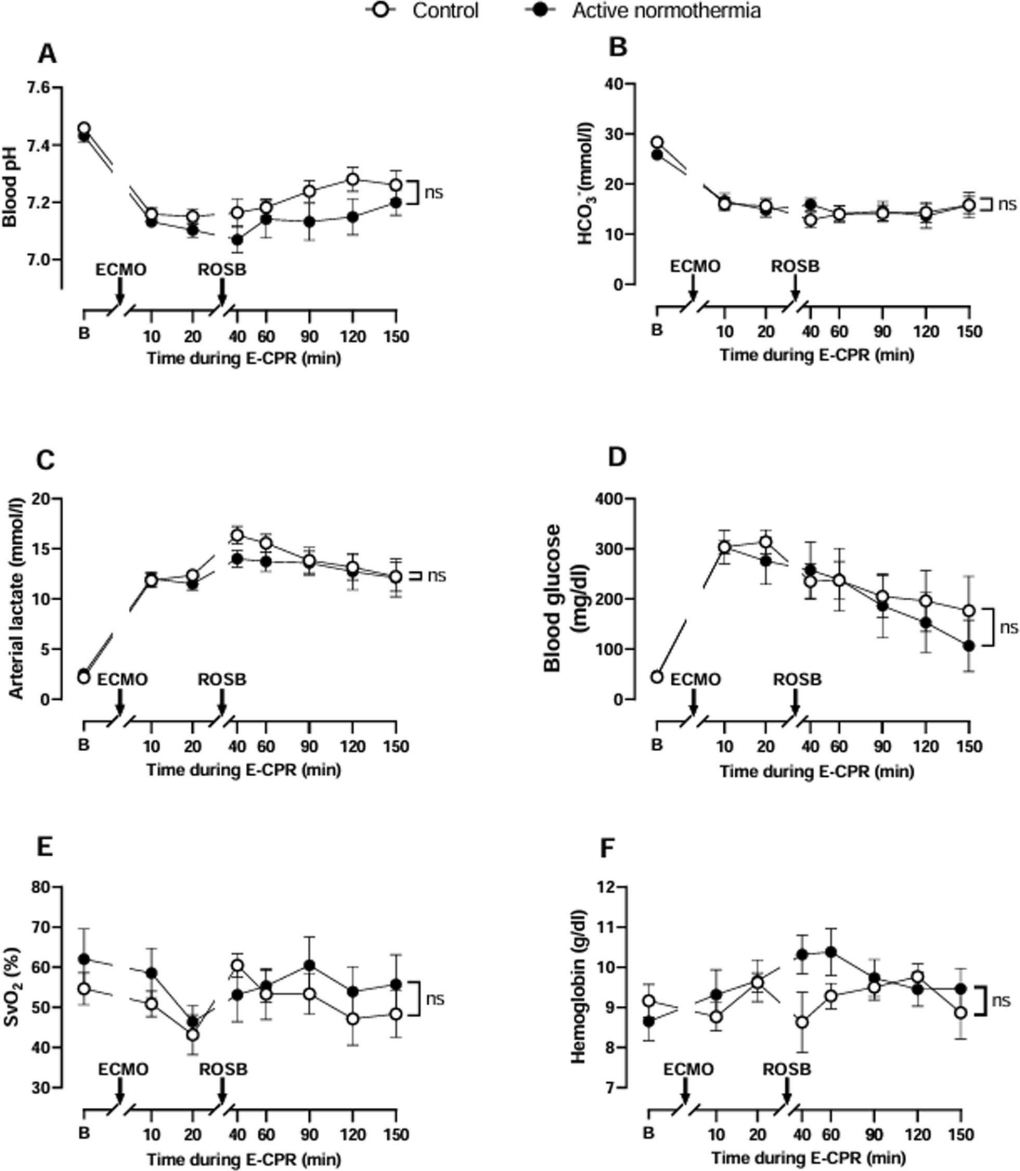
Blood gases and blood biochemistry parameters (**A**: pH, **B**: HCO3-, **C**: arterial lactate, **D**: blood glucose levels, **E**: SvO2; **F**: hemoglobin)

No significant differences were observed between groups for any of the standard blood gas parameters (pH, HCO₃⁻, SvO_2_), arterial lactate or hemoglobin levels. Although not statistically significant, pH tended to be lower in the active normothermia group after ROSB. (Fig. 6).

**Fig. 7:**
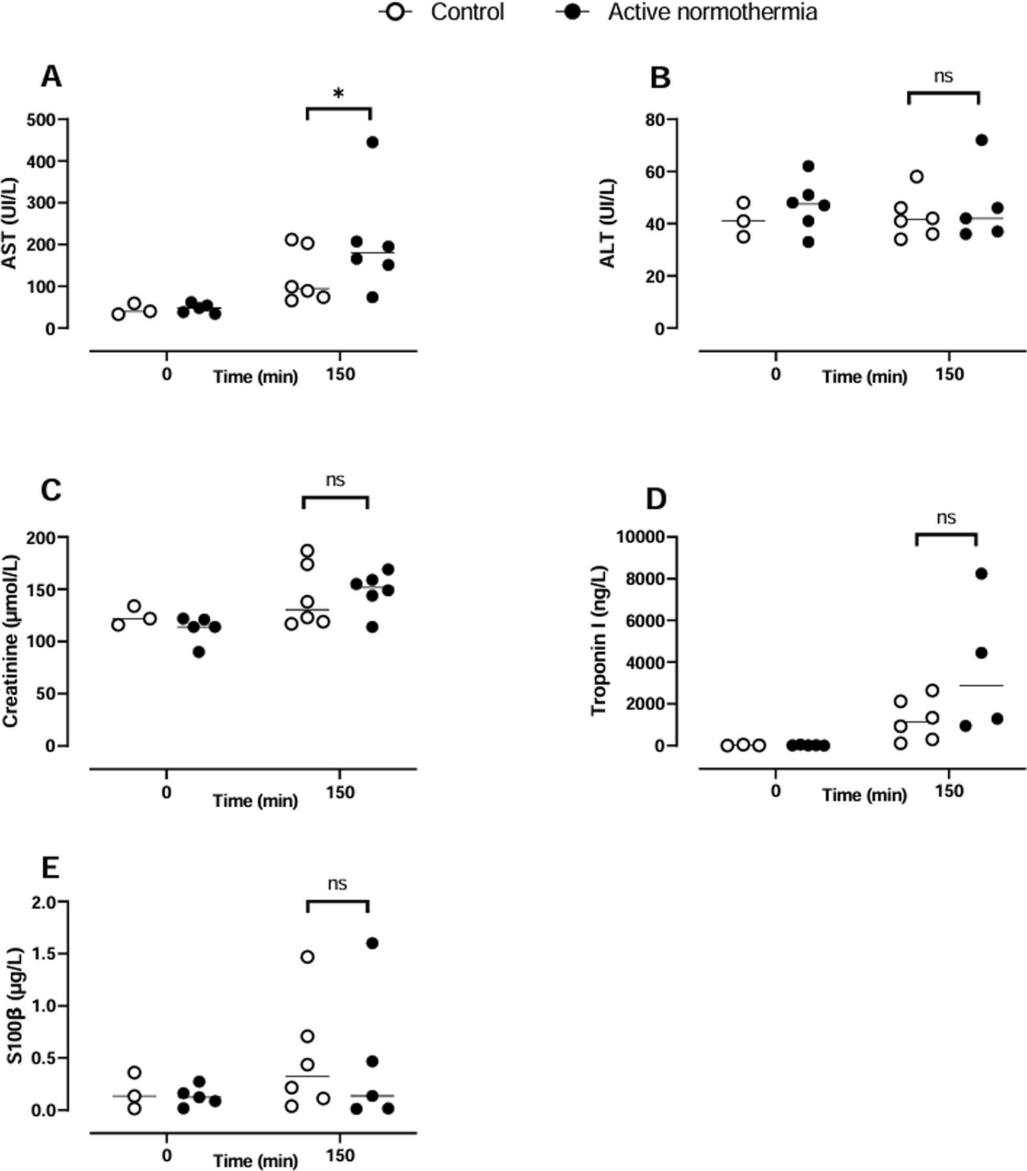
Biology parameters (**A**: AST, **B**: ALT, **C**: creatinine, **D**: troponin I, **E**: S100β). n = 6 per group. Continuous variables are expressed as mean ± SEM. Each point indicates one animal for both the total catecholamine dose and the total amount of fluids administered. *, p<0.05 for the group x time interaction effect (p=0.046) with Mixed-effects analysis. AST: Aspartate Aminotransferase; ALT: Alanine Aminotransferase; S100β: Protein S100β

No significant differences were observed between groups for blood markers of liver (ALT), cardiac (troponin I), renal (creatinine) and brain injury (Protein S100β) (Fig. 7).

## Discussion

In this present study, we compared consequences of active normothermia versus no temperature control by omitting VA-ECMO heat-exchanger during the early phase of e-CPR in a swine model of refractory cardiac arrest. We found that early active normothermia during the first hours had no beneficial short-term impact on fluid requirement or hemodynamics parameters compared to no temperature regulating device.

Despite a significant drift in core temperature between groups, no differences emerged in fluids requirements, ECMO flow, arterial pressure, or vasopressor requirements, suggesting that short-term temperature management strategy did not influence circulatory performance in the early acute phase of refractory cardiac arrest. The transient rise in right atrial pressure observed in the no temperature regulating device group may indicate a short-lived alteration in venous return, but probably without clinically relevant effect. The lack of difference in blood gases and ultra early impact on target organs biomarkers do not confirm the hypothesis that active normothermia induces very early metabolic stress or accelerates organ dysfunction [17, 18].

The literature studying the effect of temperature management on fluid requirement and hemodynamic parameters in a large model of refractory cardiac arrest is very limited. One porcine study showed the benefit of mild therapeutic hypothermia compared to controlled normothermia for the maintenance of blood pressure and cerebral oxygenation in a VA-ECMO support [19]. Contrary to our study, therapeutic hypothermia was reached at 33 °C using a procedure of active cooling. To the best of our knowledge, our present study is the first describing the hemodynamic consequences of the absence of heat-exchanger in this context. Even if we did not reach a proper hypothermia state in the group of animals without heat-exchanger and even if no significant difference was observed compared to the group benefiting from a heat-exchanger, our study allowed to reproduce a frequent clinical situation (absence of heat-exchanger on the ECMO circuit) and made it possible to better describe the physiological implications. Moreover, our study allows us to revisit the main issue of targeting temperature management in patients subjected to ECMO after a refractory cardiac arrest.

In selected cases of refractory cardiac arrest, current European guidelines support the use of VA-ECMO as a rescue therapy [20]. VA-ECMO is also recommended for selected patients experiencing out-of-hospital hypothermic cardiac arrest [21]. Despite its increasing use, evidence on temperature control strategies in e-CPR patients is inconsistent. While recent meta-analysis found no significant effect on mortality or neurological outcomes [22, 23], other studies suggest that moderate hypothermia may improve neurological recovery [24–29]. However, most eligible studies were small, retrospective, and heterogeneous. However, all the protocols use prolonged period of temperature control. In most cohorts, patients underwent targeted temperature management, and several studies have attempted to determine the optimal temperature control strategy in this population [30, 31]. However, little information is available regarding the time required to reach the target temperature or the specific methods used to rewarm hypothermic patients to normothermia in more recent trials. Finally, optimal temperature for patients undergoing e-CPR is unknown, and no data is available on temperature control strategies during and immediately after e-CPR. In this experimental study, we investigated two temperature control strategies from the start of e-CPR until the end of the 150-minute follow-up period, which is supposed to correlate in real life with the time until ICU admission.

For out-of-hospital hypothermic cardiac arrest, the ELSO guidelines recommend rewarming at a rate of approximately 5 °C per hour until a core temperature of 30 °C is reached or until return of spontaneous circulation occurs [21]. Nevertheless, both the rate and the modality of rewarming may influence neurological recovery and hemodynamic stability [32, 33]. More recently, guidelines have suggested that patients with mild hypothermia after ROSC should not undergo active rewarming to achieve normothermia [20, 34]. However, patients with refractory cardiac arrest experience longer periods of low blood flow, potentially leading to a more severe post cardiac arrest syndrome, in which the potential benefit of hypothermia would be higher. This recommendation, however, is based on low-quality evidence and has not been validated by dedicated studies; this observation motivated the present experimental investigation, due to major logistical constraint related to addition of heat-exchanger on ECMO circuit in prehospital, or interhospital transfer, setting.

A heat-exchanger warms the blood by circulating sterile water around the oxygenator membrane. Importantly, this device cannot be used in clinical routine to achieve active cooling. In patients with shock, an extracorporeal circuit operating at room temperature inevitably leads to heat loss. In the absence of a heat-exchanger, heat dissipation during VA-ECMO depends primarily on sweep gas flow and ambient temperature, rather than on ECMO blood flow itself [35]. Although it is often assumed that humans cool at a rate of approximately 1 °C per hour, this estimate remains controversial [36] A recent study evaluating VV and VA-ECMO patients managed without heat-exchanger reported that most did not develop significant hypothermia [37]. In our study, the modest temperature difference between the groups (1.4 °C) did not allow us to observe any significant physiological differences, likely due to the short follow-up period and the absence of a cooling system. Such a limited separation may not be sufficient to produce major physiologic differences, particularly within a highly dynamic e-CPR setting. However, the observed passive temperature decrease is consistent with that described above.

Extracorporeal warming systems are not without drawbacks, particularly regarding infectious risks associated with water circuits [38, 39]. Beyond additional costs, the main concern relates to hemodynamic instability. Rapid rewarming may precipitate vasodilation, worsen shock, or induce electrolyte shifts, all of which can compromise an already unstable cardiovascular state [40–42]. Despite these risks, no standardized or evidence-based protocol currently exists to guide rewarming strategies in hypothermic patients supported by ECMO. We observed the need for an increased pump speed to achieve the same VA-ECMO flow, as well as non-significant higher norepinephrine doses in the active normothermia group. Due to those signals without statistical significance, one might hypothesize that there might be a more pronounced vasoplegia and vascular leakage in the active normothermia group. The association between these findings and active rewarming remains to be further demonstrated. The absence of a rewarming device may, in fact, offer advantages in terms of simplified prehospital management. This study may lead to the conclusion that the short-term preclinical use of simplified ECMO circuits without active temperature management does not appear to result in severe hypothermia or major early hemodynamic instability over a clinically realistic transport-like timeframe.

This study presents several limitations. The number of animals was relatively small, which limits statistical power and may reduce the generalizability of the findings. Indeed, the sample size calculation was based on fluid requirement, and findings on other clinical or biological parameters should be cautiously scrutinized: the absence of statistically significant differences should not be interpreted as evidence of equivalence or absence of physiological effect. The small number of animals was in line with the current call for limitation of large animal sacrifices. The cardiac arrest model used was particularly severe, with short follow-up of 120-minutes, potentially masking more subtle physiological differences related to temperature management and precluding long-term neurological outcomes or organ damage evaluation. In addition, the heat-exchanger was a custom-made device rather than a standardized clinical system, which may affect reproducibility. However, our study demonstrates that this device may be sufficient to reach temperature targets. Core temperature was monitored exclusively through bladder measurements, a method that may lag true central temperature during rapid thermal changes. The difference in core temperature between the two groups was only 1.5 °C, which may not be enough to cause any significant impact on physiology. In fact, this difference remains clinically relevant and is more akin to a slight drop in temperature than to actual hypothermia. In addition, this temperature lag echoes the one observed in clinical practices where out-of-hospital cardiac arrest patients arrive hypothermic to the ICU; so, this difference is clinically meaningful in the clinical setting, as it would imply decision to rewarm, or not, a patient. The short 120-minute follow-up period likely prevented us from observing any significant findings regarding biomarkers, which often require longer time intervals to reflect injury evolution; this duration of protocol was explained by practical concerns of this very severe swine model. But il also reflects the time frame of prehospital e-CPR practice, where heat-exchangers are often unavailable. On the contrary, even during a short duration, hypothermia can generate arrythmias, which have not been recorded in this study. Finally, achieving the target MAP required frequent adjustments in norepinephrine dosing and fluid administration. This complex interplay between vasopressor support, preload optimization, and VA-ECMO flow made strict hemodynamic standardization a challenge and may have introduced variability. Indeed, balance between vasopressor and fluids on VA-ECMO support is a subtle situation and warrants further explorations [16, 43–45].

## Conclusion

In this experimental model of refractory cardiac arrest supported by e-CPR, active normothermia versus no temperature regulating device during the first hours of cardio-circulatory support did not result in significant differences in fluid requirements and hemodynamic parameters. The short-term preclinical use of simplified ECMO circuits without active temperature management does not appear to result in severe hypothermia or major early hemodynamic instability over a clinically realistic transport-like timeframe. Larger studies will be necessary to determine whether specific temperature control strategies can meaningfully influence outcomes in cardiac arrest patients treated with e-CPR.

## Data Availability

All data are available on reasonable request to the corresponding author.

## References

[1] Soar J, Böttiger BW, Carli P, Couper K, Deakin CD, Djärv T et al (2021) European Resuscitation Council Guidelines 2021: Adult advanced life support. Resuscitation 161:115–151 33773825 10.1016/j.resuscitation.2021.02.010

[2] Supady A, Bělohlávek J, Combes A, Hutin A, Lorusso R, MacLaren G et al (2025) Extracorporeal cardiopulmonary resuscitation for refractory cardiac arrest. Lancet Respir Med 13(9):843–56 40456239 10.1016/S2213-2600(25)00122-5

[3] Leroux L, Dennis-Benford NB, Bergeron A, Lamhaut L, Cournoyer A, Grunau B et al (2025) Impact of prehospital extracorporeal cardiopulmonary resuscitation for out-of-hospital cardiac arrest on survival with good neurological function: a systematic review and meta-analysis. Resusc Plus 24:100974 40491772 10.1016/j.resplu.2025.100974 PMC12148607

[4] Belohlavek J, Smalcova J, Rob D, Franek O, Smid O, Pokorna M et al (2022) Effect of Intra-arrest Transport, Extracorporeal Cardiopulmonary Resuscitation, and Immediate Invasive Assessment and Treatment on Functional Neurologic Outcome in Refractory Out-of-Hospital Cardiac Arrest: A Randomized Clinical Trial. JAMA 327(8):737–747 35191923 10.1001/jama.2022.1025 PMC8864504

[5] Taccone FS, Minini A, Avalli L, Alm-Kruse K, Annoni F, Bougouin W et al (2024) Impact of extracorporeal cardiopulmonary resuscitation on neurological prognosis and survival in adult patients after cardiac arrest: An individual pooled patient data meta-analysis. Resuscitation 202:110357 39142468 10.1016/j.resuscitation.2024.110357

[6] Kang JK, Darby Z, Bleck TP, Whitman GJR, Kim BS, Cho SM (2024) Post-Cardiac Arrest Care in Extracorporeal Cardiopulmonary Resuscitation. Crit Care Med 52(3):483–494 37921532 10.1097/CCM.0000000000006102 PMC10922987

[7] Cronberg T, Greer DM, Lilja G, Moulaert V, Swindell P, Rossetti AO (2020) Brain injury after cardiac arrest: from prognostication of comatose patients to rehabilitation. Lancet Neurol 19(7):611–22 32562686 10.1016/S1474-4422(20)30117-4

[8] Moreau A, Levy B, Annoni F, Lorusso R, Su F, Belliato M et al (2023) The use of induced hypothermia in extracorporeal membrane oxygenation: A narrative review. Resusc Plus 13:100360 36793940 10.1016/j.resplu.2023.100360 PMC9922920

[9] Lüsebrink E, Binzenhöfer L, Kellnar A, Scherer C, Schier J, Kleeberger J et al (2022) Targeted Temperature Management in Postresuscitation Care After Incorporating Results of the TTM2 Trial. J Am Heart Assoc Cardiovasc Cerebrovasc Dis 11(21):e026539 10.1161/JAHA.122.026539 PMC9673653 36285786

[10] Lamhaut L, Hutin A, Puymirat E, Jouan J, Raphalen JH, Jouffroy R et al (2017) A Pre-Hospital Extracorporeal Cardio Pulmonary Resuscitation (ECPR) strategy for treatment of refractory out hospital cardiac arrest: An observational study and propensity analysis. Resuscitation 117:109–117 28414164 10.1016/j.resuscitation.2017.04.014

[11] Lunz D, Philipp A, Petermichl W, Graf B, Lubnow M, Schmid C et al (2026) Prehospital initiation of extracorporeal life support for refractory out-of-hospital cardiac arrest-results of a prospective observational study. Crit Care 19(1):163 10.1186/s13054-026-05958-2 PMC13064238 41857661

[12] Nolan JP, Sandroni C, Cariou A, Cronberg T, D’Arrigo S, Haywood K et al (2025) European Resuscitation Council and European Society of Intensive Care Medicine Guidelines 2025 Post-Resuscitation Care. Resuscitation. 215

[13] Hutin A, Lamhaut L, Lidouren F, Kohlhauer M, Mongardon N, Carli P et al (2016) Early coronary reperfusion facilitates return of spontaneous circulation and improves cardiovascular outcomes after ischemic cardiac arrest and extracorporeal resuscitation in pigs. J Am Heart Assoc 5(12):e004588 28007740 10.1161/JAHA.116.004588 PMC5210433

[14] Levy Y, Hutin A, Lidouren F, Polge N, Fernandez R, Kohlhauer M et al (2021) Targeted high mean arterial pressure aggravates cerebral hemodynamics after extracorporeal resuscitation in swine. Crit Care Lond Engl 25(1):369 10.1186/s13054-021-03783-3 PMC8590749 34774087

[15] Levy Y, Hutin A, Polge N, Lidouren F, Fernandez R, Kohlhauer M et al (2022) HEAD AND THORAX ELEVATION PREVENTS THE RISE OF INTRACRANIAL PRESSURE DURING EXTRACORPOREAL RESUSCITATION IN SWINE. Shock 58(3):236–240 35959782 10.1097/SHK.0000000000001971

[16] Jendoubi A, Lidouren F, Watanabe N, San Geroteo J, Mouri N, Goutchtat R et al (2026) Deleterious effects of liberal fluid therapy during extracorporeal cardiopulmonary resuscitation in a porcine model of prolonged cardiac arrest. J Am Heart Assoc 15(2):e042711 41532520 10.1161/JAHA.125.042711 PMC12919492

[17] Ruttmann E, Weissenbacher A, Ulmer H, Müller L, Höfer D, Kilo J et al (2007) Prolonged extracorporeal membrane oxygenation-assisted support provides improved survival in hypothermic patients with cardiocirculatory arrest. J Thorac Cardiovasc Surg 134(3):594–600 17723804 10.1016/j.jtcvs.2007.03.049

[18] Debaty G, Maignan M, Perrin B, Brouta A, Guergour D, Trocme C et al (2016) Deep Hypothermic Cardiac Arrest Treated by Extracorporeal Life Support in a Porcine Model: Does the Rewarming Method Matter? Acad Emerg Med Off J Soc Acad Emerg Med 23(6):665–673 10.1111/acem.12893 26728797

[19] Ostadal P, Mlcek M, Kruger A, Horakova S, Skabradova M, Holy F et al (2013) Mild therapeutic hypothermia is superior to controlled normothermia for the maintenance of blood pressure and cerebral oxygenation, prevention of organ damage and suppression of oxidative stress after cardiac arrest in a porcine model. J Transl Med 11:124 23688243 10.1186/1479-5876-11-124 PMC3679736

[20] Soar J, Böttiger BW, Carli P, Jiménez FC, Cimpoesu D, Cole G et al (2025) European Resuscitation Council guidelines 2025 adult advanced life support. Resuscitation 215(Suppl 1):110769 41117572 10.1016/j.resuscitation.2025.110769

[21] Cools E, Swol J, Wanscher M, Brugger H, Pasquier M, McIntosh S et al (2025) ELSO 2025 Narrative Guideline on the Use of ECMO for Accidental Hypothermia. ASAIO J 71(11):865 41128452 10.1097/MAT.0000000000002557

[22] Huang M, Shoskes A, Migdady I, Amin M, Hasan L, Price C et al (2022) Does Targeted Temperature Management Improve Neurological Outcome in Extracorporeal Cardiopulmonary Resuscitation (ECPR)? J Intensive Care Med 37(2):157–167 34114481 10.1177/08850666211018982

[23] Wang J, Zhang H, Wang T, Liu G, Teng Y, Wang J et al (2025) What’s the optimal temperature control strategy in patients receiving ECPR after cardiac arrest? A network meta-analysis. Am J Emerg Med 87:74–81 39509999 10.1016/j.ajem.2024.11.001

[24] Suverein MM, Delnoij TSR, Lorusso R, Brandon Bravo Bruinsma GJ, Otterspoor L, Elzo Kraemer CV et al (2023) Early extracorporeal CPR for refractory out-of-hospital cardiac arrest. N Engl J Med 388(4):299–309 36720132 10.1056/NEJMoa2204511

[25] Rob D, Farkasovska K, Kreckova M, Smid O, Kavalkova P, Macoun J et al (2024) Effect of intra-arrest transport, extracorporeal cardiopulmonary resuscitation and immediate invasive assessment in refractory out-of-hospital cardiac arrest: a long-term follow-up of the Prague OHCA trial. Crit Care Lond Engl 28(1):125 10.1186/s13054-024-04901-7 PMC11022382 38627823

[26] Yannopoulos D, Bartos J, Raveendran G, Walser E, Connett J, Murray TA et al (2020) Advanced reperfusion strategies for patients with out-of-hospital cardiac arrest and refractory ventricular fibrillation (ARREST): a phase 2, single centre, open-label, randomised controlled trial. Lancet (London, England) 396(10265):1807–16 33197396 10.1016/S0140-6736(20)32338-2 PMC7856571

[27] Belohlavek J, Smalcova J, Rob D, Franek O, Smid O, Pokorna M et al (2022) Effect of Intra-arrest Transport, Extracorporeal Cardiopulmonary Resuscitation, and Immediate Invasive Assessment and Treatment on Functional Neurologic Outcome in Refractory Out-of-Hospital Cardiac Arrest: A Randomized Clinical Trial. JAMA. 22 févr 2022;327(8):737–47. 10.1001/jama.2022.1025 PMC8864504 35191923

[28] Hsu CH, Meurer WJ, Domeier R, Fowler J, Whitmore SP, Bassin BS et al (2021) Extracorporeal Cardiopulmonary Resuscitation for Refractory Out-of-Hospital Cardiac Arrest (EROCA): Results of a Randomized Feasibility Trial of Expedited Out-of-Hospital Transport. Ann Emerg Med 78(1):92–101 33541748 10.1016/j.annemergmed.2020.11.011 PMC8238799

[29] Scquizzato T, Bonaccorso A, Swol J, Gamberini L, Scandroglio AM, Landoni G et al (2023) Refractory out-of-hospital cardiac arrest and extracorporeal cardiopulmonary resuscitation: a meta-analysis of randomized trials. Artif Organs 47(5):806–16 36929354 10.1111/aor.14516

[30] Levy B, Girerd N, Amour J, Besnier E, Nesseler N, Helms J et al (2022) Effect of Moderate Hypothermia vs Normothermia on 30-Day Mortality in Patients With Cardiogenic Shock Receiving Venoarterial Extracorporeal Membrane Oxygenation: A Randomized Clinical Trial. JAMA 327(5):442–453 35103766 10.1001/jama.2021.24776 PMC8808325

[31] Levy B, Girerd N, Duarte K, Antoine ML, Monzo L, Ouattara A et al (2024) Hypothermia in patients with cardiac arrest prior to ECMO-VA: insight from the HYPO-ECMO trial. Resuscitation 200:110235 38762081 10.1016/j.resuscitation.2024.110235

[32] Linardi D, Walpoth B, Mani R, Murari A, Tessari M, Hoxha S et al (2020) Slow versus fast rewarming after hypothermic circulatory arrest: effects on neuroinflammation and cerebral oedema. Eur J Cardiothorac Surg 58(4):792–800 32408343 10.1093/ejcts/ezaa143

[33] Saczkowski R, Kuzak N, Grunau B, Schulze C (2021) Extracorporeal life support rewarming rate is associated with survival with good neurological outcome in accidental hypothermia. Eur J Cardiothorac Surg 59(3):593–600 33230533 10.1093/ejcts/ezaa385

[34] ANZCOR (2026) Guideline 11.8 – Temperature Control after Cardiac Arrest, 2026

[35] Mojoli F, Bianzina S, Caneva L, Tavazzi G, Mongodi S, Pozzi M et al (2015) Temperature Monitoring During Ecmo: An in Vitro Study. Intensive Care Med Exp 3(Suppl 1):A506

[36] Sessler DI (2001) Complications and Treatment of Mild Hypothermia. Anesthesiology 95(2):531 11506130 10.1097/00000542-200108000-00040

[37] Hoyler M, Baidya J, Rippon B, Debois W, Srivastava A, Iannacone E et al (2024) Temperature outcomes without heater cooler units in adult patients supported with extracorporeal membrane oxygenation: a retrospective cohort study. Perfusion 39(7):1380–7 37559410 10.1177/02676591231195694

[38] Ditommaso S, Giacomuzzi M, Memoli G, Zotti CM (2021) Real-time PCR, the best approaches for rapid testing for Mycobacterium chimaera detection in heater cooler units and extracorporeal membrane oxygenation. Perfusion 36(6):626–629 33054627 10.1177/0267659120963878

[39] Baker MA, Rhee C, Tucker R, Vaidya V, Holtzman M, Seethala RR et al (2022) Ralstonia pickettii and Pseudomonas aeruginosa Bloodstream Infections Associated With Contaminated Extracorporeal Membrane Oxygenation Water Heater Devices. Clin Infect Dis Off Publ Infect Dis Soc Am 75(10):1838–1840 10.1093/cid/ciac379 35594555

[40] Thoresen M, Whitelaw A (2000) Cardiovascular changes during mild therapeutic hypothermia and rewarming in infants with hypoxic-ischemic encephalopathy. Pediatrics 106(1 Pt 1):92–99 10878155 10.1542/peds.106.1.92

[41] Gebauer CM, Knuepfer M, Robel-Tillig E, Pulzer F, Vogtmann C (2006) Hemodynamics Among Neonates With Hypoxic-Ischemic Encephalopathy During Whole-Body Hypothermia and Passive Rewarming. Pediatrics 117(3):843–850 16510666 10.1542/peds.2004-1587

[42] Saleh M, Barr TA (2005) The Impact of Slow Rewarming on Inotropy, Tissue Metabolism, and After Drop of Body Temperature in Pediatric Patients. J Extra Corpor Technol 37(2):173–179 16117455 PMC4682534

[43] Jendoubi A, de Roux Q, Ribot S, Vanden Bulcke A, Miard C, Tiquet B et al (2024) Fluid management in adult patients undergoing venoarterial extracorporeal membrane oxygenation: a scoping review. J Crit Care 86:155007 39709803 10.1016/j.jcrc.2024.155007

[44] Jendoubi A, De Roux Q, Lê MP, Magnoni S, Ghaleh B, Tissier R et al (2025) Fluid therapy during and after cardiopulmonary resuscitation for nontraumatic cardiac arrest: a systematic review of evidence from preclinical and clinical studies. Shock 63(3):363–70 40016801 10.1097/SHK.0000000000002519

[45] Jendoubi A, de Roux Q, Ribot S, Desauge V, Betbeder T, Picard L et al (2025) Optimising fluid therapy during venoarterial extracorporeal membrane oxygenation: current evidence and future directions. Ann Intensive Care 15(1):32 40106084 10.1186/s13613-025-01458-8 PMC11923310

